# Evaluation of an interprofessional school-based health centre: A multimethods study

**DOI:** 10.1093/pch/pxaf123

**Published:** 2025-12-18

**Authors:** Susan Tang, Kathleen Morgan, Jessy Gatete, Mary Hendrickson, Hugues Plourde, Noa Hitterman, Reina Remman, David Estok, Geoffrey Dougherty, Matthew Donlan, David D’Arienzo

**Affiliations:** Faculty of Agricultural and Environmental Sciences, School of Human Nutrition, McGill University, Montreal, Quebec, Canada; Department of Nutrition, Heart & Hands Pediatric Clinic, Montreal, Quebec, Canada; Faculty of Medicine and Health Sciences, McGill University, Montreal, Quebec, Canada; Faculty of Agricultural and Environmental Sciences, School of Human Nutrition, McGill University, Montreal, Quebec, Canada; Faculty of Agricultural and Environmental Sciences, School of Human Nutrition, McGill University, Montreal, Quebec, Canada; Faculty of Medicine and Health Sciences, School of Physical & Occupational Therapy, McGill University, Montreal, Quebec, Canada; Faculty of Medicine and Health Sciences, School of Communication Sciences and Disorders, McGill University, Montreal, Quebec, Canada; Department of Nutrition, Heart & Hands Pediatric Clinic, Montreal, Quebec, Canada; Department of Pediatrics, Montreal Children's Hospital, McGill University, Montreal, Quebec, Canada; Department of Pediatrics, Montreal Children's Hospital, McGill University, Montreal, Quebec, Canada; Department of Pediatrics, Montreal Children's Hospital, McGill University, Montreal, Quebec, Canada

**Keywords:** Multidisciplinary, School clinic, Health care access, Health promotion, Advocacy

## Abstract

**Objectives:**

School-based health centres (SBHCs) aim to improve access to care, especially among socially vulnerable children. Interprofessional SBHCs are emerging, though their evaluations remain limited. We evaluated an interprofessional SBHC in a low-income community.

**Methods:**

The evaluation of the SBHCs was guided by the RE-AIM framework. The multimethods design included (i) a document analysis and (ii) a retrospective chart review of SBHC patients from September 2021 to May 2024.

**Results:**

The SBHC team comprised of paediatricians, a dietitian, occupational therapist, speech language pathologist, and a nurse working alongside school staff and community providers. After 98 clinic half-days, 760 appointments were scheduled across 153 patients, with a nonattendance rate of 12%. Care needs extended beyond preventative medicine for 80% of children, with mental health conditions being the most common diagnoses.

**Conclusion:**

This evaluation demonstrates that interprofessional SBHCs are feasible and offer a scalable model for addressing the complex health needs of socially vulnerable children.

## Introduction

School-based health centres (SBHCs) aim to improve access to healthcare for children and youth, especially those in underserved communities (1). Originally developed to provide onsite primary care, SBHCs increasingly serve populations with complex health needs that extend beyond basic medical services (2,3). By integrating multiple health professions, interprofessional SBHCs centralize services and improve access to care (3). They are well positioned to address the medical, developmental, and psychosocial needs of socially vulnerable students, yet evaluations of such models remain limited.

The location of SBHCs reduces barriers to accessing healthcare, such as transportation, time off work, and mistrust of healthcare, while offering care in a familiar setting (4). SBHCs have been associated with improved attendance, mental health, vaccination uptake, reduced emergency visits, and narrower income-based disparities in access (3). Over time, SBHCs have expanded from a primary-care model to broader interprofessional platforms. In the USA, approximately 3900 SBHCs operate, with 71% reporting expanded teams in 2021 to 2022 compared with 41% in 2016 to 2017 (1). Common additions include behavioural health, oral health, dietetics, and rehabilitation services (3). While many evaluations confirm the benefits of primary-care SBHCs, there is a lack of evaluations of interprofessional SBHCs that integrate allied health professionals and a lack of evaluations of SBHCs in the Canadian context (5,6). This study evaluates an interprofessional SBHC in a Canadian low-socioeconomic community using the RE-AIM framework to characterize its service delivery, quantify reach and effectiveness, and examine sustainability (7).

## METHODS

### Study design

This study employed a multimethods design guided by RE-AIM framework. We conducted (i) a document analysis of SBHC protocols and policies and (ii) a retrospective chart review of all children aged 0–18 years who received medical, dietetics, occupational therapy (OT), speech language pathology (SLP), or nursing services between September 1, 2021, and May 17, 2024. Ethics approval was granted by the McGill University Institutional Review Board (A02-E17-24A).

### Setting

**Figure 1.**
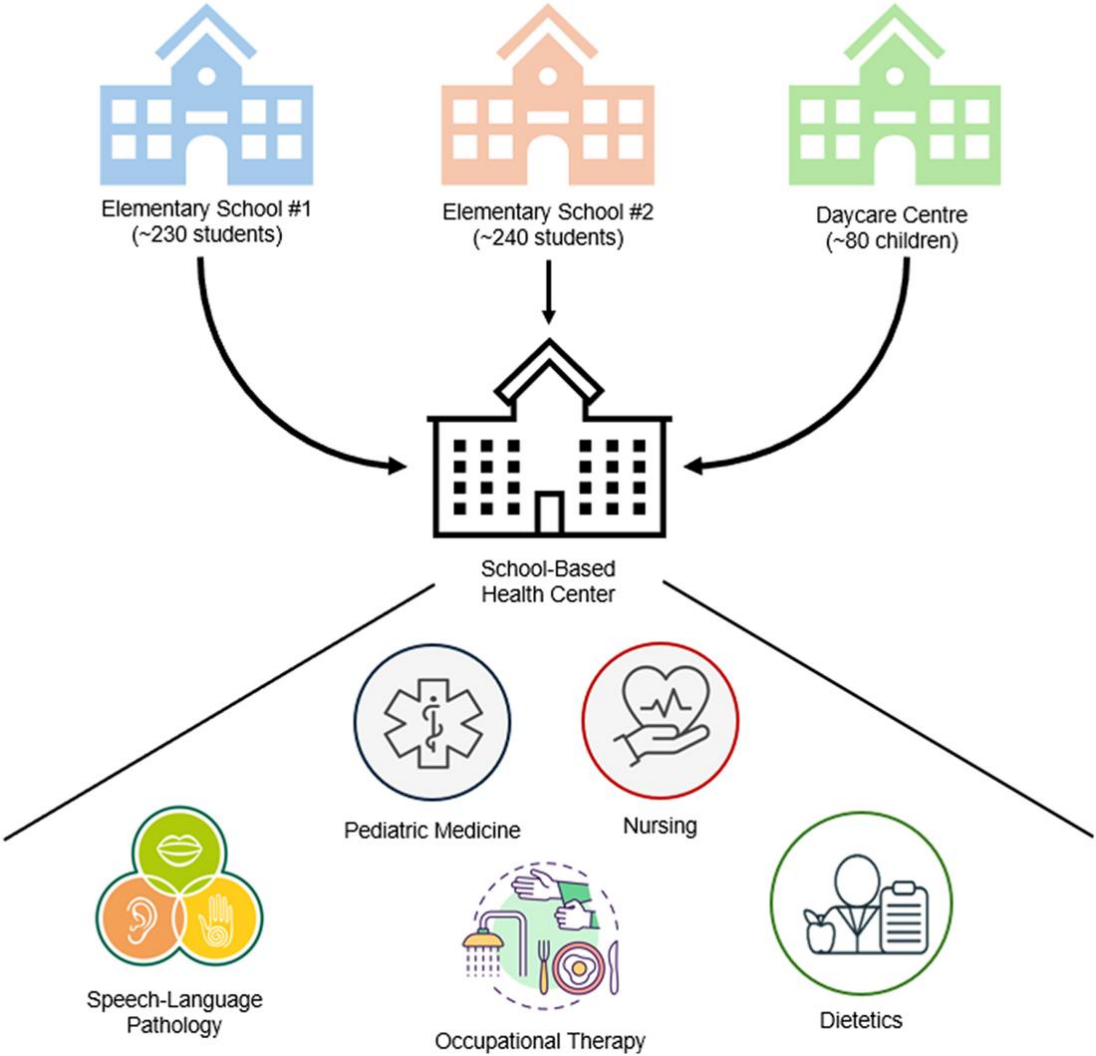
Organizational structure of the school-based health centre services.

The SBHC under evaluation is built in a Canadian elementary school and serves a low-income community, with approximately 20% of the population living in poverty, high rates of immigrants and visible minorities, and low rates of attachment to primary-care services (8,9). The interprofessional team comprises two paediatricians (with residents), a dietitian, an occupational therapist, a speech language pathologist, and a clinical nurse, phased in variably between 2021 and 2024. The clinic operates one half-day per week during the school year, with all professions present the same day. The SBHC operates with an open referral process, accepting referrals from all, including teachers, school staff members, and parents/caregivers (Figure 1).

### Data analysis

Document analysis was conducted to evaluate the program's adoption and implementation and identify interprofessional activities, roles, and coordination mechanisms, validated through discussion with clinic leadership. Retrospective chart reviews were used to evaluate the program's reach, effectiveness, and maintenance. Metrics for each RE-AIM domain reflected established measures used in evaluations of health systems interventions (7,10,11). Adoption was assessed by integration of the SBHC into school workflows and referral processes. Implementation was evaluated by exploring services delivered and frequency of services. Reach was measured by the number and characteristics of children served, visit volumes, and attendance rates. Effectiveness was assessed through diagnoses, clinical issues addressed, and interventions provided. Maintenance was evaluated by the extent to which clinic operations and interprofessional activities were sustained over time. Data extracted included patient demographics, service use, diagnoses made, clinical interventions, and health promotion activities. Diagnoses, made by the clinicians at the time of the patient assessment based on history, laboratory/imaging investigations, and use of standardized tools (where appropriate), were independently categorized by two clinicians per discipline; disagreements were resolved by consensus. Descriptive statistics were reported as mean (standard deviation [SD] [12]), median (interquartile range [IQR]), and frequency (proportions). Analyses were conducted in SAS Institute v9.4.

## RESULTS

### Service delivery (adoption and implementation)

Across the study period, physicians provided 98 clinic half-days; dietetics had 48 clinic half-days, OT 40 clinic half-days, SLP 30 clinic half-days, and nursing 12 clinic half-days (clinic frequency varied based on professionals' start date and funding available for each service). In addition to clinical services, health promotion activities were developed in response to requests from school staff. This included sessions on healthy eating, personal hygiene, and sleep hygiene, among other relevant topics. Additionally, interprofessional meetings were instituted to support shared assessment and coordinated planning. The clinic nurse, who served as the liaison between the SBHC and school staff and provided direct patient care, care coordination, and follow-up for medical/mental health conditions, also participated in the school's weekly resource team meetings, to discuss struggling students and how the SBHC can support the patient and school staff.

### Reach

A total of 153 children were assessed at the SBHC (mean age 7.3 years, SD 4.0; 50% female) (Table 1). Across the 5 health professions, 41 children (27%) were seen by 2 or more professions and 18 (12%) by 3 or more. Across 760 scheduled appointments, 502 (66%) were for medicine, 76 (10%) for dietetics, 82 (11%) for OT, 100 (13%) for SLP, and 48 (6%) for nursing (Table 2). The overall attendance rate was 88%, with OT recording the highest nonattendance rate (19%). The most common reason for referral is presented in Table 3. Most children (80%) required care beyond preventive services, defined as patients needing care beyond health supervision visits (well-child visits), age-appropriate screenings, immunizations, anticipatory guidance, and counselling on healthy living. Additionally, 80% of the children assessed at the SBHC had an intervention for new or existing health concerns (13). Fifty-five health promotion events were organized, reaching 949 children and families. The dietitian led 42 nutrition workshops for 649 students and 5 parent/family sessions (48 attendances), and the nurse delivered classroom sessions on bullying and sexual health to 252 students.

**Table 1.** School-based health centre (SBHC) users’ baseline characteristics, by profession

	Number (%)				
	Medicine	Dietetics	OT	SLP	Nursing
	n (%)	n (%)	n (%)	n (%)	n (%)
	(n = 142)	(n = 17)	(n = 8)	(n = 16)	(n = 10)
Age (years)*					
<1	13 (9.2)	0 (0)	0 (0)	0 (0)	0 (0)
1–6	47 (33.1)	5 (29.5)	3 (37.5)	2 (12.5)	5 (50.0)
7–12	70 (49.3)	9 (52.9)	5 (62.5)	14 (87.5)	3 (30.0)
13–18	12 (8.5)	3 (17.6)	0 (0)	0 (0)	2 (20.0)
Sex					
Male	71 (50)	7 (41.2)	5 (62.5)	12 (75.0)	5 (50.0)
Female	71 (50.0)	10 (58.8)	3 (37.5)	4 (25.0)	5 (50.0)
Referred by					
SBHC health provider	N/A	13 (76.5)	5 (62.5)	3 (18.8)	10 (100)
School health provider	N/A	0 (0)	1 (12.5)	10 (62.5)	0 (0)
Teacher/school staff member	N/A	3 (17.6)	1 (12.5)	3 (18.8)	0 (0)
Parent or caregiver	N/A	1 (5.9)	1 (12.5)	0 (0)	0 (0)

^*^Age at first appointment.

OT, occupational therapy; SLP, speech language pathology; N/A, not available.

**Table 2.** School-based health centre appointment summary: number of total visits, completed visits, no-shows, and cancellations, by profession

	Number of visits scheduled (%)			
	Medicine	Dietetics	OT	SLP
	n (%)	n (%)	n (%)	n (%)
Total Appointments scheduled	502 (100)	76 (100)	82 (100)	100 (100)
Completed visits	449 (89.4)	63 (82.9)	66 (80.5)	91 (91.0)
No-shows	14 (2.8)	5 (6.6)	9 (11)	9 (9.0)
Cancellations	39 (7.8)	8 (10.5)	7 (8.5)	0 (0)

OT, occupational therapy; SLP, speech language pathology.

**Table 3.** Most common reasons for referral to each school-based health centre profession

Reasons for referral	Number of patients n (%)
To medical services	
Unmet medical needs	57 (40.1)
Behaviour concerns	48 (33.8)
To dietetics services	
High BMI	7 (41.2)
Picky eating	5 (29.4)
To occupational therapy services	
Fine and gross motor concerns	3 (37.5)
Only fine motor concerns	2 (25.0)
To speech language pathology services	
Speech and sound disorder	7 (43.8)
Language disorder	6 (37.5)
To nursing services	
Coordination of care for complex cases	10 (100.0)

BMI, body mass index.

### Effectiveness

Across all patients seen at the SBHC, the most common medical diagnoses made at the SBHC included attention deficit/hyperactivity disorder (n = 27, 18%), obesity (n = 24, 16%), and generalized anxiety disorder (n = 16, 11%), constipation (n = 15, 10%), and autism (n = 13, 9%). Among 17 dietetics patients, 22 nutrition-related problems were identified, including inadequate fibre (41%), vitamin D (41%), calcium (35%), and fluid intake (24%). The median number of nutrition problems per patient was 2 (IQR 1–6). Among eight OT patients, 18 issues were recorded, most commonly handwriting (63%), gross motor coordination (50%), and visual-motor integration (38%), with a median of 6 per patient (IQR 4.75–6). Among 16 SLP patients, disorders included speech sound (44%), language (38%), and dyslexia (19%), with a median of 1 per patient (IQR 1–1). Interventions reflected these needs. The dietitian provided a median of 3 interventions per patient (IQR 2–4), most often nutrition counselling on balanced eating (77%). OT provided a median of 2.5 interventions (IQR 1–4), most often home programming (75%). SLP delivered a median of 3 interventions (IQR 2–4), primarily caregiver guidance (94%) involving education and strategies for language support (Table 4).

**Table 4.** Interventions provided by each allied health professionals at the school-based health centre provider

	Number of patients n (%)
Nutrition interventions	
Balanced eating	13 (76.5)
Division of responsibility*	8 (47.1)
Diet expansion strategies	7 (41.2)
Food skills and cooking	6 (35.3)
Dietary management of digestive symptoms	5 (29.4)
Increase or decrease intake of a specific nutrient	5 (29.4)
Coordination of care with external resources^†^	4 (23.5)
Other	3 (17.6)
Occupational therapy interventions	
Home programming	6 (75.0)
Fine motor skill development	5 (62.5)
Gross motor skill development	5 (62.5)
Executive functioning	2 (25.0)
Self-care	2 (25.0)
Emotional regulation	2 (25.0)
Speech language pathology interventions	
Caregiver implementation strategies^‡^	15 (93.8)
Language development	11 (68.8)
Literacy	10 (62.5)
Speech sound production	8 (50.0)
Fluency	2 (12.5)
Communication skills	1 (6.3)

^*^Including education and strategies on the roles of caregivers versus children surrounding eating and mealtime, developed by Ellyn Satter.

^†^Including access to food assistance.

^‡^Caregiver implementation strategies are defined as the caregiving delivering therapeutic techniques or environmental modifications to address SLP concerns.

SLP, speech language pathology.

### Maintenance

Several processes became embedded into routine operations. The SBHC continued to run a weekly half-day clinic with open referral pathways. Interprofessional clinical meetings were held every 2 weeks, with alternating administrative meetings involving community organizations. The nurse served as a standing member of the school resource team, ensuring follow-up for medically or socially complex cases. Health promotion activities also continued in response to school requests.

## DISCUSSION

This evaluation describes an interprofessional SBHC that serves students from low-socioeconomic communities. Over 98 clinic half-days, 153 children attended 760 appointments with 88% attendance; 27% received care from 2 or more professions, and 80% required services beyond prevention.

Attendance rates at a SBHC were greater than national averages. The medical nonattendance rate was 10%, lower than 11–20% typically observed in primary-care clinics and general paediatric clinics across both Canada and the United States (14–16). Dietetics and SLP nonattendance rates (17% and 9%, respectively) were also lower than outpatient comparators from the United States (29% and 20%) (17,18). These findings suggest that colocation within a trusted school setting and the ability to coordinate services enhanced access and reliability. OT services had lower patient volume and higher nonattendance rates of −20%, comparable to community OT (19). Reasons for lower patient volumes and higher nonattendance rates among the OT services include referral uncertainty among teachers and burdensome weekly OT appointments for families (20,21).

The interprofessional model enabled identification and management of common nutrition and developmental concerns that single-service clinics may miss (5,6). Dietetic findings reflected national concerns with fibre, vitamin D, and calcium intake, while OT findings mirrored reports that handwriting difficulties are the most common deficit in school-aged children (22,23). Interventions such as caregiver-implemented strategies, which is where the patient's caregiver delivers therapeutic techniques or environmental modifications to address underlying concerns, demonstrate how colocated allied health can translate assessment into immediate treatment (24). In this study, low allied health volumes likely reflect limited clinic days and staffing availability rather than rather than lack of demand, as 80% of children required care beyond prevention.

School-linked allied health services elsewhere are often constrained by large caseloads and fragmented practice (25). Embedding these services in an interprofessional SBHC offers complementary capacity, shorter access pathways, and tighter integration with educators. Expanded allied health presence has been associated with better academic and socio-emotional outcomes and stronger collaboration between education and health sectors (3).

Health promotion workshops extended reach beyond referred students, with 949 attendees. This aligns with evidence that school-based programs led by embedded professionals improve health knowledge and behaviours (26). Interprofessional SBHCs can tailor these activities to community priorities, supporting prevention and well-being at scale.

This study has limitations. Data were retrospective and may be incomplete. Referral pathways shifted during the study and allied health volumes were constrained by clinic days and awareness. Sociodemographic characteristics of individual patients served were not available; therefore, community characteristics were presented. Developmental diagnoses requiring long-term observation may have been underidentified. Finally, findings derive from a single centre, limiting generalizability.

## CONCLUSION

An interprofessional SBHC reached students with unmet medical, nutritional, motor, and speech language needs while extending health promotion activities to a wider community. This model proved effective in addressing diverse concerns within a socially vulnerable population, supported by collaboration across professions and integration with the school environment. Attendance rates compared favourably to outpatient benchmarks, highlighting potential advantages of access and coordination inherent to school-based care. Next steps should include evaluation of patient-reported outcomes, comparative, and economic analyses (including return on investment) and refinement of referral and scheduling pathways to enhance reach, efficiency, and sustainability. Interprofessional SBHCs represent a promising approach to reducing inequities and addressing the complex health needs of underserved children.

## References

[1] Soleimanpour S, Cushing K, Christensen J, et al, Findings from the 2022 National Census of School-Based Health Centers. United States of America: School-Based Health Alliance, 2023.

[2] Dunfee MN . School-based health centers in the United States: Roots, reality, and potential. J Sch Health 2020;90(8):665–70. 32567122 10.1111/josh.12914

[3] Kjolhede C, Lee AC. School-based health centers and pediatric practice. Pediatrics 2021;148(4):e2021053758. 34544844 10.1542/peds.2021-053758

[4] Boudreaux M, Chu J, Lipton BJ. School-based health centers, access to care, and income-based disparities. JAMA Netw Open 2023;6(9):e2334532. 37721750 10.1001/jamanetworkopen.2023.34532 PMC10507491

[5] D'Arienzo D, Xu S, Shahid A, et al Evaluating the feasibility and outcomes of a resident-led school-based pediatric clinic. Paediatr Child Health 2023;28(6):349–56. 37744759 10.1093/pch/pxad016 PMC10517241

[6] Freeman S, Sgro M, Wormsbecker AE, et al Feasibility study on the Model Schools Paediatric Health Initiative pilot project. Paediatr Child Health 2013;18(7):361–6. 24421711 PMC3804636

[7] Glasgow RE, Vogt TM, Boles SM. Evaluating the public health impact of health promotion interventions: The RE-AIM framework. Am J Public Health 1999;89(9):1322–7. 10474547 10.2105/ajph.89.9.1322 PMC1508772

[8] City of Montreal CG , Verdun: Territorial Analysis 2018-2019. Quebec, Canada: Centraid, 2019.

[9] City of Montreal . Planification de Verdun 2023-2028—Notre quartier inclusif et solidaire. 2023. <https://portail-m4s.s3.montreal.ca/pdf/rapport_annuel_verdun_2023_planif_strategique_vf.pdf> (Accessed August 11, 2025).

[10] Glasgow RE, Harden SM, Gaglio B, et al RE-AIM planning and evaluation framework: Adapting to new science and practice with a 20-year review. Front Public Health 2019;7:64. 30984733 10.3389/fpubh.2019.00064 PMC6450067

[11] Glasgow RE, Estabrooks PE. Pragmatic applications of RE-AIM for health care initiatives in community and clinical settings. Prev Chronic Dis 2018;15:E02. 29300695 10.5888/pcd15.170271 PMC5757385

[12] Putman CE, Baum S, Nagy EC. Academy of radiology research begins operations. Radiology 1996;198(1):292. 8539397 10.1148/radiology.198.1.8539397

[13] Committee on Practice and Ambulatory Medicine . 2025 recommendations for preventive pediatric health care: Policy statement. Pediatrics 2025;155(5):e2025071066. 39914362 10.1542/peds.2025-071066

[14] Dantas LF, Fleck JL, Cyrino Oliveira FL, Hamacher S. No-shows in appointment scheduling—A systematic literature review. Health Policy 2018;122(4):412–21. 29482948 10.1016/j.healthpol.2018.02.002

[15] Huang Y, Hanauer DA. Patient no-show predictive model development using multiple data sources for an effective overbooking approach. Appl Clin Inform 2014;5(3):836–60. 25298821 10.4338/ACI-2014-04-RA-0026 PMC4187098

[16] Shahab I, Meili R. Examining non-attendance of doctor's appointments at a community clinic in Saskatoon. Can Fam Physician 2019;65(6):e264–e8. 31189640 PMC6738377

[17] Carnino JM, Bayly H, Mwaura AM, et al Exploring the appointment factors affecting pediatric patients with swallow disorders: Implications for speech and language pathology attendance. Int J Pediatr Otorhinolaryngol 2023;175:111778. 37956556 10.1016/j.ijporl.2023.111778 PMC11365569

[18] Jones MK, O'Connell NS, Skelton JA, Halvorson EE. Patient characteristics associated with missed appointments in pediatric subspecialty clinics. J Healthc Qual 2022;44(4):230–9. 35302524 10.1097/JHQ.0000000000000341

[19] Smith R, Gallego G. Parents' ability to access community health occupational therapy services in a disadvantaged area: A proof of concept study. Aust Occup Ther J 2021;68(1):54–64. 32986879 10.1111/1440-1630.12699

[20] Truong V, Thompson-Hodgetts S. An exploration of teacher perceptions toward occupational therapy and occupational therapy practices: A scoping review. J Occup Ther Sch Early Interv 2017;10:121–136.

[21] Fairbairn M, Davidson I. Teachers' perceptions of the role and effectiveness of occupational therapists in schools. Can J Occup Ther 1993;60(4):185–91.

[22] de Oliveira Borba PL, Pereira BP, de Souza JRB, Lopes RE. Occupational therapy research in schools: A mapping review. Occup Ther Int 2020;2020:5891978. 32904500 10.1155/2020/5891978 PMC7455833

[23] Government of Canada . Do Canadian children and adolescents meet their nutrient requirements through food intake alone? 2012. <https://www.canada.ca/en/health-canada/services/food-nutrition/food-nutrition-surveillance/health-nutrition-surveys/canadian-community-health-survey-cchs/canadian-adolescents-meet-their-nutrient-requirements-through-food-intake-alone-health-canada-2012.html> (Accessed August 13, 2025).

[24] Lawler K, Taylor NF, Shields N. Outcomes after caregiver-provided speech and language or other allied health therapy: A systematic review. Arch Phys Med Rehabil 2013;94(6):1139–60. 23187042 10.1016/j.apmr.2012.11.022

[25] O'Donoghue C, O'Leary J, Lynch H. Occupational therapy services in school-based practice: A pediatric occupational therapy perspective from Ireland. Occup Ther Int 2021;2021:6636478. 34220382 10.1155/2021/6636478 PMC8221888

[26] Shackleton N, Jamal F, Viner RM, et al School-based interventions going beyond health education to promote adolescent health: Systematic review of reviews. J Adolesc Health 2016;58(4):382–96. 27013271 10.1016/j.jadohealth.2015.12.017

